# Time for change: Transitions between HIV risk levels and determinants of behavior change in men who have sex with men

**DOI:** 10.1371/journal.pone.0259913

**Published:** 2021-12-09

**Authors:** Maartje G. J. Basten, Daphne A. van Wees, Amy Matser, Anders Boyd, Ganna Rozhnova, Chantal den Daas, Mirjam E. E. Kretzschmar, Janneke C. M. Heijne

**Affiliations:** 1 Julius Center for Health Sciences and Primary Care, University Medical Center Utrecht, Utrecht University, Utrecht, The Netherlands; 2 Department of Infectious Diseases, Research and Prevention, Public Health Service of Amsterdam, Amsterdam, The Netherlands; 3 Stichting HIV Monitoring, Amsterdam, The Netherlands; 4 BioISI – Biosystems & Integrative Sciences Institute, Faculdade de Ci*ê*ncias, Universidade de Lisboa, Lisboa, Portugal; 5 Center for Infectious Diseases Control, National Institute for Public Health and the Environment, Bilthoven, The Netherlands; 6 Aberdeen Health Psychology Group, Institute of Applied Health Sciences, Aberdeen, Scotland; UNSW Australia, AUSTRALIA

## Abstract

As individual sexual behavior is variable over time, the timing of interventions might be vital to reducing HIV transmission. We aimed to investigate transitions between HIV risk levels among men who have sex with men (MSM), and identify determinants associated with behavior change. Participants in a longitudinal cohort study among HIV-negative MSM (Amsterdam Cohort Studies) completed questionnaires about their sexual behavior during biannual visits (2008–2017). Visits were assigned to different HIV risk levels, based on latent classes of behavior. We modelled transitions between risk levels, and identified determinants associated with these transitions at the visit preceding the transition using multi-state Markov models. Based on 7,865 visits of 767 participants, we classified three risk levels: low (73% of visits), medium (22%), and high risk (5%). For MSM at low risk, the six-month probability of increasing risk was 0.11. For MSM at medium risk, the probability of increasing to high risk was 0.08, while the probability of decreasing to low risk was 0.33. For MSM at high risk, the probability of decreasing risk was 0.43. Chemsex, erection stimulants and poppers, high HIV risk perception, and recent STI diagnosis were associated with increased risk at the next visit. High HIV risk perception and young age were associated with decreasing risk. Although the majority of MSM showed no behavior change, a considerable proportion increased HIV risk. Determinants associated with behavior change may help to identify MSM who are likely to increase risk in the near future and target interventions at these individuals, thereby reducing HIV transmission.

## Introduction

Timing of preventive interventions is essential to reduce transmission of infectious diseases, and is particularly important for pre-exposure prophylaxis (PrEP) use to reduce HIV transmission. PrEP is a highly effective HIV prevention drug [1] that was introduced in the Netherlands in 2015, and became available via STI clinics in 2019. For effective use of PrEP, it is crucial to take periods of behavior related to high HIV risk into account, as timely initiation of PrEP could prevent HIV infections, and timely termination of PrEP could prevent medication overuse, lack of adherence, and high costs [2]. In the Netherlands, around 700 persons are currently diagnosed with HIV each year. Men who have sex with men (MSM) accounted for 66% of newly diagnosed HIV infections in 2018 [3]. Several preventive interventions have been found effective in reducing HIV transmission, including condom use, regular testing, and treatment with antiretroviral therapy (ART) as prevention (e.g., Undetectable = Untransmittable (U = U)) [4, 5]. These interventions have likely contributed to the decrease in number of diagnoses in the Netherlands observed since 2008 [3]. Despite the implementation of these interventions, HIV transmission among MSM continues, and PrEP use may further reduce HIV transmission.

In the Netherlands, HIV-negative MSM are eligible for PrEP use when they meet at least one of the following criteria: reporting to have had condomless anal intercourse (AI) with a male partner with unknown HIV status or known HIV-positive partner with a detectable viral load, at least one syphilis or anal sexually transmitted infection (STI) diagnosis, or to have used post-exposure prophylaxis (PEP) in the past six months [6]. HIV prevention, such as PrEP initiation, is currently tailored towards current behavior or behavior in the recent past, but is not tailored to expected changes in behavior and specific periods of sexual behavior associated with high HIV risk in the (near) future [4]. This means that HIV-negative MSM who are at the onset of increasing HIV risk may be viewed as low risk based on their past behavior, and do not meet the PrEP eligibility criteria at present, whereas timely initiation of PrEP may be especially important to prevent HIV transmission in these individuals.

Previous research identified predictors of sexual behavior associated with HIV risk. For example, determinants that are associated with condomless AI and high number of partners include the use of erection stimulants and poppers, chemsex, group sex [7–13], increased STI incidence, younger age (<35 years) [14]. In addition, a steady partnership may be associated with decreasing risk, such as decreased number of partners [13]. Also, HIV risk perception is associated with sexual behavior [14–16]. For example, high HIV risk perception may indicate that an individual is aware of the increased HIV risk associated with their current sexual behavior. At the same time, this awareness may provide motivation to decrease risk in the future. However, few studies have examined which determinants are associated with imminent changes in sexual behavior. A further reduction in HIV transmission may be achieved by designing interventions that target MSM just before they enter a period of high HIV risk.

The aim of the current study was to examine transitions between risk levels based on sexual behavior among MSM at risk of HIV, using longitudinal data from the Amsterdam Cohort Studies (ACS) [17]. We applied multi-state Markov models to examine transitions between different levels of risk behavior across six-monthly intervals, in line with Dutch guidelines for six-monthly STI/HIV testing among MSM. Furthermore, we aimed to identify determinants of imminent changes in sexual behavior that go beyond the PrEP eligibility criteria, and more specifically, of increasing and decreasing HIV risk.

## Materials and methods

### Study population and procedure

The ACS started in 1984, and is an open, ongoing prospective cohort study on the epidemiology, sexual behavior, psychosocial determinants, course of infection, and pathogenesis of HIV among MSM in Amsterdam [17]. The ACS was approved by the Medical Ethics Committee of the Academic Medical Center, University of Amsterdam, Amsterdam, the Netherlands (MEC 07/182). Participation is voluntary and written informed consent is obtained from every participant at intake. Men are eligible for participation if they live in or around Amsterdam and had sex with other men in the six months prior to recruitment (see also [14, 18]). Participants were recruited by “convenience sampling” (e.g. brochures at the STI clinic, advertisements in the gay scene) and “chain referral sampling” (i.e. participants recruited by other participants). Recruitment was limited to young MSM under 30 years of age during several time periods, to prevent aging of the cohort. Participants did not receive any incentive or compensation for participation, but the six-monthly consultations and STI/HIV testing in the ACS is provided free of charge. MSM who agreed to participate completed a self-administered questionnaire about their sexual behavior and related psychosocial determinants in the preceding six months. Also, at each visit, MSM were tested for HIV and other STI, including syphilis, gonorrhea, and chlamydia (for test details see [14, 18]).

The current study comprised MSM who visited the ACS at least twice between October 2008 and July 2017. MSM were included in the present study if they were HIV negative at their first visit during this study period. Follow-up continued until HIV seroconversion, last ACS visit, death, or July 2017, whichever occurred first.

### Sexual behavior risk score

We used a composite sexual behavior risk score predictive of HIV seroconversion to define HIV risk levels, which has been developed in previous work [13]. Briefly, this risk score was based on sexual behavior assessed at every visit, and included detailed information about anal intercourse (AI) with casual partners in the preceding six months, including numbers of AI partners, condom use during AI, and number of condomless AI partners with an unknown or positive HIV status. All information was reported separately for insertive and receptive AI, and for three types of casual partners: one-night stands (‘someone you met by chance and had sex with only once’), multiple-time casual partners (‘someone you met by chance on several occasions and had sex with on these occasions’) and sex buddies (‘someone you intentionally contact on a regular basis to have sex with’). Condom use was reported on a 5-point Likert scale from 1 ‘always used a condom’ to 5 ‘never used a condom’. Information on whether the participant engaged in AI during group sex (yes/no) was also included in the risk score. The risk score predicted HIV acquisition fairly accurately in the ACS (AUC = 0.77).

### Potential determinants of behavior change

Potential determinants associated with behavior change in the near future that are not included as PrEP eligibility criteria are age (i.e., <35 years, or ≥35 years old), non-anal STI in the past six months, steady partnership (yes/no), chemsex (yes/no), erection stimulants and poppers (yes/no), and high HIV risk perception (yes/no). To compare the association of these determinants on imminent behavior change with one of the current PrEP eligibility criteria, anal STI or syphilis in the past six months was examined as a determinant of behavior change as well. Anal or non-anal STI included both gonorrhea and chlamydia diagnoses. Chemsex was defined as use of Gamma-Butyrolactone (GBL), Gamma-Hydroxybutyrate (GHB), mephedrone, methamphetamine, ketamine, amphetamine, cocaine or ecstasy (XTC) during sexual intercourse in the past six months. Erection stimulants and poppers were defined as using one or both of these sexual performance enhancing drugs during sex in the past six months. Perceived risk to have acquired HIV in the past six months was measured on a 7-point Likert scale from 1 ‘impossible’ to 7 ‘very high’ [16], and high HIV risk perception was defined as all scores higher than the median (score 3–7).

In order to determine whether sexual behavior associated with HIV risk increased or decreased over the follow-up period, calendar time was also included as potential determinant of behavior change. PrEP use in past six months was compared between the risk levels, but was not included as a potential determinant, because data on PrEP use was not assessed in the ACS until the second half of 2015, and PrEP use was limited until the national PrEP program started in 2019. Furthermore, the COVID-19 pandemic had a great influence on sexual behavior and PrEP care in 2020 and 2021, which is why the time period 2008 to 2017 was used.

### Statistical analyses

To distinguish different levels of HIV risk, we performed latent class analysis (LCA) on all visits using the previously developed sexual behavior risk score predictive of HIV seroconversion [13]. Models with one to four latent classes were estimated. To determine the number of latent classes that best fitted the data (final model), we used the following criteria: Bayesian Information Criterion (BIC) with lower values indicating better model fit, entropy with values ≥0.80 indicating good classification and Vuong-Lo-Mendell-Rubin (VLMR) likelihood ratio test with p-values < 0.05 indicating significant improvement in model fit compared to a model with one class less. Also, classes should include a substantial percentage of visits (>2%) and differ considerably in risk level [19, 20]. Based on the final model, we classified each visit in the most likely latent class risk level. Subsequently, we compared risk levels on sexual behavior characteristics, determinants possibly associated with behavior change, and PrEP use, using ANOVA for continuous variables and Chi-square test for dichotomous variables.

To examine changes in sexual behavior, transitions between risk levels were modelled as a Markov process [21]. Using a multistate time-homogenous Markov model, we modelled transition intensities between risk levels, which are defined as the instantaneous rates per year of a transition occurring, and are estimated by the probability of occupying a given state at each observed study visit. ACS visits are usually scheduled twice a year. In addition, six-monthly STI/HIV testing is recommended for MSM according to Dutch guidelines. Therefore, from the model, we obtained six-month transition probabilities that represent the likelihood of changing to a different risk level at time t+1, given the occupied state at time t. The model assumes that each transition is independent and identically distributed. These six-month transition probabilities represent mean transition probabilities across the time period between 2008 and 2017.

To identify determinants associated with transition between risk levels, we modelled the effects of potential determinants of transitions between risk levels as proportional hazards. We examined whether determinants reported at the current visit were associated with transitioning to another risk level at the next visit six months later. Visits with missing values on one or more of the potential determinants of behavior change were excluded from the model. For each determinant hazard ratios (HR) and their 95% confidence intervals (CI) of each possible transition were calculated in univariable models. All determinants were included in a multivariable model, in which the individual contribution of each determinant to change in sexual behavior was assessed. Stata version 15.1 was used for data management [22], the LCA analyses were performed in Mplus version 7.4 [23], and the multi-state Markov model was estimated by maximum likelihood methods using the ‘msm’ package in R version 3.4.1 [24].

## Results

### Sample characteristics

Between 20 October 2008 and 31 July 2017, 8,922 HIV-negative ACS visits of 833 MSM were reported. Visits without questionnaire data (n = 372), visits with missing values in the variables used to calculate the sexual behavior risk score (n = 676), and visits without follow-up data in the study period (i.e., ≤1 ACS visit during study period, n = 9) were excluded. In total, 767 MSM with 7,865 visits were included in this study (88% of all HIV-negative ACS visits), with a median number of 10 visits (IQR = 6–15) per participant (S1 Table). The majority of the study population was Dutch (81.9%), and highly educated (76.8%). At the first visit in the study period (2008–2017), mean age was 36 years (SD = 10) and mean age at sexual debut with a man was 18 (SD = 4). During follow-up, in the 767 included MSM, 37 HIV seroconversions were detected (5%).

### Risk levels

The LCA showed that models with 3 or 4 latent classes had a better model fit than a 2-class solution (i.e., higher BIC) (S2 Table). The VLMR p-value showed that the model with 4 latent classes was not a significantly better fitting model than the model with 3 latent classes. Furthermore, the smallest latent class in the 4-class solution was very small (<2%). Therefore, the model with the three-class solution was chosen (entropy value = 0.91). Based on the characteristics of the latent classes that were identified, 5,786 visits were classified as low (73%), 1,712 as medium (22%), and 367 as high risk (5%) of acquiring HIV (Table 1). The percentage of HIV seroconversions was 0.1% in intervals classified as low risk, 0.2% in medium risk, and 2.5% in high risk.

**Table 1:** Characteristics reported at visits classified as low, medium and high risk among MSM participating in the Amsterdam Cohort Studies, Amsterdam the Netherlands, between 2008 and 2017 (n = 7,865).

	Low risk n visits = 5,786	Medium risk n visits = 1,712	High risk n visits = 367
	*M* (SD)	*M* (SD)	*M* (SD)
Number of casual insertive AI partners	1.4 (4.5)	6.0 (10.0)	16.2 (16.7)
Number of casual receptive AI partners	0.4 (0.9)	5.4 (4.9)	19.5 (19.2)
	n (%)	n (%)	n (%)
Condomless AI^a^ with casual partner			
Yes	695 (12.0)	766 (44.7)	296 (80.7)
No	5,089 (88.0)	945 (55.2)	71 (19.3)
AI^a^ during group sex			
Yes	388 (6.7)	1,056 (61.7)	324 (88.3)
No	5,398 (93.3)	655 (38.3)	43 (11.7)
Age < 35			
Yes	1,687 (29.2)	442 (25.8)	77 (21.0)
No	4,099 (70.8)	1,270 (74.2)	290 (79.0)
Steady partnership			
Yes	3,929 (67.9)	999 (58.4)	202 (55.0)
No	1,855 (32.1)	712 (41.6)	162 (44.1)
Chemsex^b^			
Yes	884 (15.3)	705 (41.2)	203 (55.3)
No	4,801 (83.0)	974 (56.9)	158 (43.1)
Erection stimulants and poppers^c^			
Yes	2,106 (36.4)	1,288 (75.2)	322 (87.7)
No	3,604 (62.3)	401 (23.4)	44 (12.0)
High HIV risk perception (HIV risk perception score>2)^d^			
Yes	986 (17.0)	527 (30.8)	183 (49.9)
No	4,738 (81.9)	1,171 (68.4)	181 (49.3)
Anal bacterial STI or syphilis in past 6 months^e^			
Yes	227 (3.9)	214 (12.5)	83 (22.6)
No	5,363 (92.7)	1,483 (86.6)	281 (76.6)
Non-anal bacterial STI in past 6 months^e^			
Yes	169 (2.9)	84 (4.9)	28 (7.6)
No	5,421 (93.7)	1,613 (94.2)	336 (91.6)
	n/N (%)	n/N (%)	n/N (%)
PrEP use in past six months^f^	23/1,336 (1.7)	35/450 (7.8)	42/135 (31.1)

Note. All characteristics are significantly different between the low, medium, and high risk visits (p-values <0.001), except for age and steady partnerships between medium and high risk visits. Categories do not all add up to the total number of visits, as missing values are not shown.

^a^Insertive or receptive AI;

^b^Including GBL, GHB, mephedrone, methamphetamine, ketamine, amphetamine, cocaine or XTC;

^c^Including poppers or erectile dysfunction drugs during sex;

^d^Median HIV risk perception = 2;

^e^Based on test findings at the ACS or the STI clinic, available from October 2008 onwards;

^f^ Assessed in ACS from second half of 2015 onwards including 1,921 visits.

Abbreviations: AI = anal intercourse; M = mean; PrEP = pre-exposure prophylaxis; SD = standard deviation; STI = sexually transmitted infection.

### Behavior change

**Fig 1:**
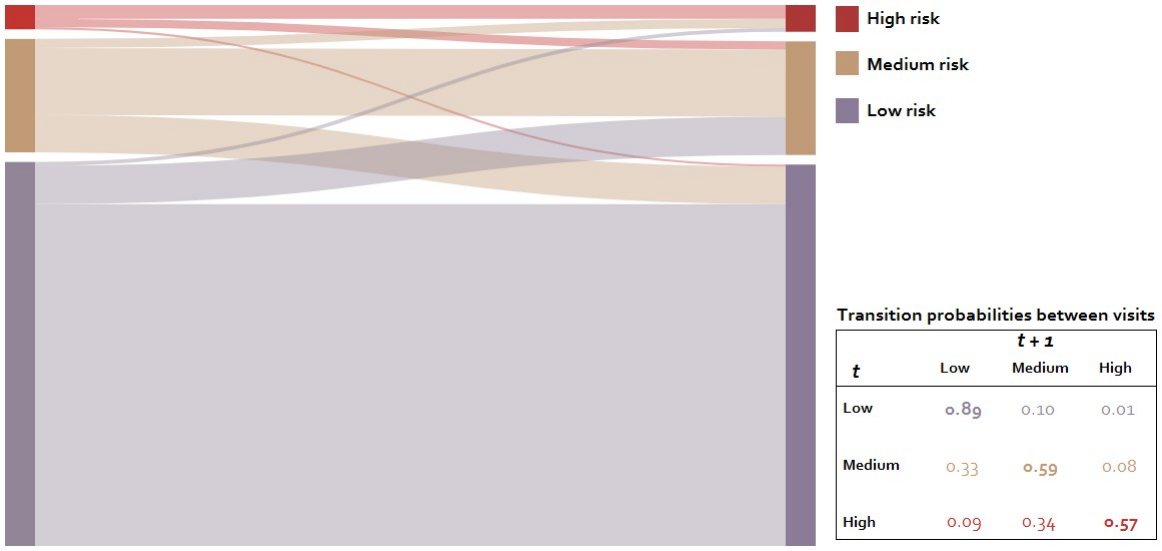
Six-month transition probabilities between visits classified as low (73%), medium (22%), and high risk (5%) among MSM participating in the Amsterdam Cohort Studies, Amsterdam the Netherlands, between 2008 and 2017 (n = 7,865). Thickness of the lines represent the group size relative to the rest of the population.

From the Markov model, we obtained the six-month transition probabilities between risk levels. In the data, moving to a high risk visit directly after a low risk visit or to a low risk visit directly after a high risk visit was very rare. Therefore, in the model it was assumed that if individuals transitioned between low risk and high risk in the data, they must have occupied medium risk level at some point during the six-month time interval. The modelled transition probabilities between visits showed that for MSM at low risk, the probability of moving to medium risk was 0.10, and of moving to high risk was 0.01 (Fig 1). For MSM at medium risk, the probability of moving to high risk was 0.08, and the probability of moving to low risk was 0.33. For MSM at high risk, the probability of moving to medium risk was 0.34, and to low risk was 0.09. MSM were more likely to remain at the same risk level at the next visit if their previous visit was classified as low risk (0.89) compared to medium (0.59) or high (0.57) risk.

We examined determinants of increasing risk level, that is moving from low-> medium and medium->high, and decreasing risk level, i.e., high->medium, and medium->low risk level in univariable and multivariable analyses (Table 2). In multivariable analysis, reporting chemsex, use of erection stimulants and poppers, high HIV risk perception, anal STI or syphilis in the past six months, and non-anal STI in the past six months was associated with increasing from low risk at the current visit to medium risk at the next visit. Reporting chemsex, high HIV risk perception, and anal STI or syphilis in the past six months was associated with increasing from medium risk at the current visit to high risk at the next visit. Calendar time was associated with all transitions towards increasing risk (low->medium, medium->high) (S3 Table), which means that sexual behavior associated with medium or high HIV risk increased over follow-up.

**Table 2:** Univariable and multivariable determinants of increasing (low to medium, or medium to high risk level) or decreasing HIV risk (high to medium, or medium to low risk level) in proportional hazards analysis among MSM participating in the Amsterdam Cohort Studies, Amsterdam the Netherlands, between 2008 and 2017 (n = 7,427 visits).

	Increasing HIV risk				Decreasing HIV risk			
	Low -> Medium		Medium -> High		High -> Medium		Medium -> Low	
	*Crude*	*Adjusted*	*Crude*	*Adjusted*	*Crude*	*Adjusted*	*Crude*	*Adjusted*
	HR (95% CI)	HR (95% CI)	HR (95% CI)	HR (95% CI)	HR (95% CI)	HR (95% CI)	HR (95% CI)	HR (95% CI)
Age < 35	1.14 (0.94–1.38)	1.18 (0.96–1.46)	0.91 (0.60–1.38)	0.82 (0.52–1.28)	1.22 (0.80–1.87)	1.05 (0.65–1.70)	**1.24** (1.02–1.50)	**1.28** (1.03–1.59)
Steady partnership	**0.77** (0.64–0.92)	**0.81** (0.66–0.98)	**0.62** (0.44–0.88)	0.70 (0.48–1.02)	**0.52** (0.37–0.75)	**0.60** (0.39–0.90)	**0.79** (0.66–0.94)	**0.80** (0.66–0.97)
Chemsex	**2.58** (2.12–3.14)	**1.78** (1.43–2.21)	**1.79** (1.27–2.53)	**1.77** (1.21–2.60)	1.20 (0.84–1.71)	1.48 (0.96–2.28)	0.98 (0.82–1.18)	1.02 (0.83–1.25)
Erection stimulants and poppers	**2.50** (2.08–3.00)	**2.05** (1.68–2.50)	1.13 (0.70–1.82)	0.93 (0.55–1.57)	**0.46** (0.28–0.74)	**0.47** (0.25–0.85)	0.88 (0.72–1.07)	0.95 (0.76–1.17)
High HIV risk perception	**2.12** (1.74–2.59)	**1.75** (1.41–2.18)	**2.15** (1.52–3.04)	**2.03** (1.41–2.93)	1.02 (0.72–1.46)	0.97 (0.66–1.42)	**1.34** (1.10–1.62)	**1.32** (1.07–1.62)
Anal STI or syphilis in past 6 months	**2.92** (2.13–4.01)	**1.95** (1.38–2.74)	**2.11** (1.32–3.38)	**2.03** (1.22–3.38)	1.45 (0.93–2.26)	1.47 (0.91–2.38)	1.08 (0.80–1.45)	0.97 (0.71–1.33)
Non-anal STI in past 6 months	**2.42** (1.59–3.68)	**1.87** (1.18–2.96)	1.01 (0.48–2.12)	1.03 (0.45–2.37)	0.81 (0.40–1.66)	1.03 (0.47–2.26)	1.34 (0.88–2.03)	1.19 (0.73–1.92)

Notes. Visits with missings were excluded (n = 438, 6%). Of all low risk visits, 6.3% was excluded from multivariable analysis due to missing values on one or more of the potential determinants of behavior change (n = 364). This percentage missing was significantly higher compared to 3.6% of medium visits that were excluded due to missing values (n = 62), and 3.3% of high risk visits (n = 12) (p-value<0.001). Hazard ratios are calculated relative to staying at the same risk level. Hazard ratios are shown in bold when the p-value is smaller than 0.05. Abbreviations: CI = confidence interval; HR = hazard ratio; STI = sexually transmitted infection.

High HIV risk perception and young age at the current visit were associated with decreasing from medium to low risk at the next visit. MSM who were in a steady partnership were more likely to remain in the same risk level (Table 2).

## Discussion

The majority of MSM remained in the same risk level during the entire follow-up period, but a considerable proportion of MSM at low and medium risk increased to higher risk levels. Furthermore, overall HIV risk based on sexual behavior increased over time. Reporting chemsex, use of erection stimulants and poppers, high HIV risk perception, anal STI or syphilis in the past six months, and non-anal STI in the past six months were associated with increasing risk over time. High HIV risk perception and young age were associated with decreasing risk.

To our knowledge, this is one of the first longitudinal studies among MSM into determinants associated with increasing or decreasing HIV risk six months later. The main strengths of our study are the large sample size, and the availability of extensive longitudinal data spanning almost a decade (2008–2017) on both sexual behavior and other HIV/STI-related risk factors in a population of HIV-negative MSM. There were some limitations. First, the majority of the ACS study population are highly educated Dutch MSM, which means that the results may not be generalizable to the entire MSM population in the Netherlands. Nevertheless, demographics and sexual behavior as reported in the ACS has been found to be comparable to larger studies among MSM in the Netherlands, such as the Schorer monitor [25] and the "Men & Sexuality" survey [26], and to the MSM population visiting STI clinics in the Netherlands [27]. Second, since PrEP effectively reduces HIV risk [2], MSM at medium or high risk using PrEP are actually at low risk of acquiring HIV. Nonetheless, even though PrEP users are at low risk of acquiring HIV, determinants associated with behavior change are useful to improve timing of interventions, such as terminating PrEP, or increasing STI testing uptake when PrEP users remain in the medium or high risk level in terms of sexual behavior. Third, only MSM who reported to have had a male partner in the past six months were eligible to participate in the ACS, which could mean that MSM in the ACS are higher risk compared to the general MSM population. However, most participants are followed up for many years including time intervals in which they did not have a male sexual partner. These time intervals were classified as low risk, which means that the results may also reflect changes in behavior of MSM who are not sexually active. Last, we cannot rule out that changes in risk level may to some extend be explained by misclassification of risk level due to uncertainties of the LCA method. However, the entropy value was very high, which means that the risk of misclassification was very limited.

In the present study, we found that MSM who were at medium or high risk at some point during follow-up were more likely to switch between risk levels over time compared to MSM at low risk. This is in agreement with previous longitudinal studies that showed that sexual behavior over time was most variable in MSM with a high number of contacts [9], high contact rates [8], and in MSM reporting more condomless receptive and insertive AI [28, 29]. In contrast, another study among young MSM aged 18–24 years in the United States, showed that there was high variability in sexual behavior over time in all risk groups [30]. This difference may be explained by differences in the study populations, as we included MSM aged 18–77 years. We found that changing behavior over time was associated with young age, which might explain the higher variability in behavior found in the study of Wong et al [30]. Another possible explanation for the difference is that the risk groups in the study of Wong et al were based on either the number of partners or on condom use [30], whereas the risk levels in our study were based on a combination of number of partners, condom use and several other factors.

Calendar time was a determinant of increasing HIV risk which means that sexual behavior associated with high HIV risk increased during the study period (2008–2017). This is in agreement with other studies exploring sexual behavior in a similar time period [31–33]. The increase in sexual behavior associated with high STI and HIV risk may be related to important developments in terms of HIV prevention and treatment. The introduction of biomedical interventions improving treatment and prevention of HIV, such as ART and PrEP, may have had a large impact on HIV risk perception and subsequent behavior [16]. As PrEP effectively diminishes HIV risk, HIV risk perception may decrease, which might result in increased insertive and receptive condomless AI and STI risk among PrEP users [10, 14–16, 34]. Interestingly, in the current study, high HIV risk perception was associated with increasing risk, as well as decreasing risk. High HIV perception predicting increasing risk may reflect awareness of MSM that their behavior is changing towards higher HIV risk, whereas high HIV risk perception predicting decreasing risk may reflect awareness of their recent high risk behavior motivating them to decrease risk.

Anal STI or syphilis in the past six months is already included in the PrEP eligibility criteria, which can be confirmed by our results. Non-anal STI in the past six months was also associated with increasing risk. An explanation for these results may be that MSM who previously received STI diagnosis and treatment may have reduced fear, shame and stigma regarding an STI diagnosis [35, 36] (e.g., various non-invasive treatment options), and might not be inhibited by an STI diagnosis and continue to increase their risk. In addition to (non-)anal STI or syphilis, other determinants of increasing risk identified in this study were not having a steady partner, chemsex, use of erection stimulants and poppers, and high HIV risk perception. All these determinants may help to identify MSM who are likely to increase risk in a short period of time, which could be used to improve the timing of PrEP initiation.

Most previous studies have focused on exploring HIV risk factors to identify MSM eligible for initiation of PrEP. However, there are no specific criteria for stopping with PrEP described in the national guidelines, except for medical reasons, such as kidney damage. We showed that MSM at high risk are likely to decrease their risk over six-monthly intervals. Determinants associated with decreasing risk in MSM at high risk, such as high HIV risk perception, young age, and not having a steady partner, could be used to determine whether an individual should stop using PrEP or change from daily to event-driven PrEP use. This will prevent medication overuse, lack of adherence, and high costs [2]. Thus, determinants associated with decreasing or increasing risk could be helpful to identify MSM who are likely to change behavior within a short period of time, consequently enabling improved timing of interventions. For example, timely initiation or termination of PrEP use, targeting MSM who are likely to increase risk with behavior change interventions, or optimize STI/HIV testing intervals (e.g., after three months instead of after six months).

The methods in this study could be applied to other infectious diseases as well. Risk behavior plays an important role in the prevention of many infectious diseases, not only for sexually transmitted diseases. Identifying determinants of changing behavior, for example towards decreased adherence to COVID-19 measures (e.g., physical distancing, hygiene measures), might be very useful to identify individuals that should be targeted with increased infection prevention measures.

Future research should focus on assessing the impact of improved timing of interventions on HIV prevalence, using mathematical modelling. In the Netherlands, PrEP became available in 2015, but the national PrEP program started from July 2019 onwards, and PrEP use is expected to increase in the coming years [37]. Studies could focus on exploring short-term and long-term changes in sexual behavior among PrEP users, and their consequences for STI risk. Also, identifying behavior change interventions that can achieve risk-reducing behavior more effectively among MSM at high risk may be important to reduce STI transmission among PrEP users.

To conclude, the majority of ACS visits among HIV-negative MSM between 2008 and 2017 was classified as low risk. The majority of MSM showed no behavior change, but a considerable proportion of MSM increased HIV risk. Determinants of behavior change could be used for an early identification of MSM who are likely to change their behavior in the near future. Targeting interventions at MSM prior to entering a period of increased risk may help to reduce HIV transmission.

## Supporting information

S1 Table Sample characteristics. (DOCX)

S2 Table Fit statistics for latent class analysis to define levels of HIV risk based on sexual behavior. (DOCX)

S3 Table Univariable and multivariable determinants of increasing (low to medium, or medium to high risk level) or decreasing HIV risk (high to medium, or medium to low risk level) in proportional hazards analysis with calendar time, and excluding age, among MSM participating in the Amsterdam Cohort Studies, Amsterdam the Netherlands, between 2008 and 2017 (n = 7,427 visits). (DOCX)
