# Supplementary material for: Time for change: Transitions between HIV risk levels and determinants of behavior change in men who have sex with men
Source: PLoS One. 2021 Dec 9;16(12):e0259913. doi: 10.1371/journal.pone.0259913 (PMC8659368; doi:10.1371/journal.pone.0259913)
Supplement: S1 Table — (DOCX) [file pone.0259913.s001.docx]

**S1 Table. Sample characteristics**

|  | N=767  n visits=7,865 |
| --- | --- |
| Number of visits, median (IQR) | 10 (6-15) |
| Migration background^a*^ |  |
| Dutch, n (%) | 593 (77.3) |
| Non-Dutch, n (%) | 131 (17.1) |
| Education level^b*^ |  |
| Low/medium, n (%) | 178 (23.2) |
| High, n (%) | 589 (76.8) |
| Age^c^, mean (SD) | 36.3 (9.7) |
| Age sexual debut with a man, mean (SD) | 18.2 (4.1) |
| HIV seroconversions during follow-up |  |
| Yes, n (%) | 37 (4.8) |
| No, n (%) | 730 (95.2) |

* Not all categories add up to the total number of participants, as missing values are not shown.

**^a^**Dutch=participant and at least one parent was born in the Netherlands; non-Dutch= participant and at least one parent born outside the Netherlands (first-generation migrant), or participant was born in the Netherlands and both parents were born outside the Netherlands (second-generation migrant) [39].

^b^Low/medium education level=no college degree; high education level=college or university degree^.^

^c^Reported on the first visit within the study period 2008-2017.

Abbreviations: HIV=human immunodeficiency virus; IQR=interquartile range; SD=standard deviation.
