# Supplementary material for: Time for change: Transitions between HIV risk levels and determinants of behavior change in men who have sex with men
Source: PLoS One. 2021 Dec 9;16(12):e0259913. doi: 10.1371/journal.pone.0259913 (PMC8659368; doi:10.1371/journal.pone.0259913)
Supplement: S2 Table — (DOCX) [file pone.0259913.s002.docx]

**S2 Table. Fit statistics for latent class analysis to define levels of HIV risk based on sexual behavior**

| Number of classes | BIC | Entropy | VLMR p-value | % of smallest class |
| --- | --- | --- | --- | --- |
| 1 | 17849 | - | - | - |
| 2 | 15445 | .88 | .022 | 16.0% |
| 3 | 13924 | .91 | .014 | 4.8% |
| 4 | 13017 | .91 | .115 | 1.9% |

Note. Model is based on N = 7,865 visits.

Abbreviations: BIC= Bayesian information criterion; VLMR= Vuong-Lo-Mendell-Rubin likelihood ratio test of model fit.
