# Supplementary material for: Time for change: Transitions between HIV risk levels and determinants of behavior change in men who have sex with men
Source: PLoS One. 2021 Dec 9;16(12):e0259913. doi: 10.1371/journal.pone.0259913 (PMC8659368; doi:10.1371/journal.pone.0259913)
Supplement: S3 Table — (DOCX) [file pone.0259913.s003.docx]

**S3 Table. Univariable and multivariable determinants of increasing (low to medium, or medium to high risk level) or decreasing HIV risk (high to medium, or medium to low risk level) in proportional hazards analysis with calendar time, and excluding age, among MSM participating in the Amsterdam Cohort Studies, Amsterdam the Netherlands, between 2008 and 2017 (n=7,427 visits)**

|  | Increasing HIV risk | | | | Decreasing HIV risk | | | |
| --- | --- | --- | --- | --- | --- | --- | --- | --- |
|  | Low -> Medium | | Medium -> High | | High -> Medium | | Medium -> Low | |
|  | *Crude* | *Adjusted* | *Crude* | *Adjusted* | *Crude* | *Adjusted* | *Crude* | *Adjusted* |
|  | HR (95% CI) | HR (95% CI) | HR (95% CI) | HR (95% CI) | HR (95% CI) | HR (95% CI) | HR (95% CI) | HR (95% CI) |
| Calendar time (years) | **1.04**  (1.00-1.08) | 1.01  (0.96-1.05) | **1.18**  (1.09-1.28) | **1.20**  (1.09-1.31) | 1.06  (0.99-1.15) | **1.10**  (1.01-1.20) | 0.99  (0.95-1.03) | 0.98  (0.94-1.02) |
| Steady partnership | **0.77**  (0.64-0.92) | **0.81**  (0.67-0.98) | **0.62**  (0.44-0.88) | **0.68**  (0.46-1.00) | **0.52**  (0.37-0.75) | **0.56**  (0.37-0.86) | **0.79**  (0.66-0.94) | **0.80**  (0.65-0.96) |
| Chemsex | **2.58**  (2.12-3.14) | **1.77**  (1.43-2.20) | **1.79**  (1.27-2.53) | **1.84**  (1.25-2.70) | 1.20  (0.84-1.71) | **1.54**  (1.01-2.37) | 0.98  (0.82-1.18) | 1.02  (0.83-1.26) |
| Erection stimulants and poppers | **2.50**  (2.08-3.00) | **2.02**  (1.66-2.46) | 1.13  (0.70-1.82) | 0.90  (0.54-1.52) | **0.46**  (0.28-0.74) | **0.45**  (0.25-0.82) | 0.88  (0.72-1.07) | 0.92  (0.75-1.15) |
| High HIV risk perception | **2.12**  (1.74-2.59) | **1.74**  (1.40-2.16) | **2.15**  (1.52-3.04) | **1.79**  (1.23-2.61) | 1.02  (0.72-1.46) | 0.88  (0.60-1.31) | **1.34**  (1.10-1.62) | **1.33**  (1.08-1.64) |
| Anal STI or syphilis in past 6 months | **2.92**  (2.13-4.01) | **1.99**  (1.42-2.80) | **2.11**  (1.32-3.38) | **1.96**  (1.16-3.30) | 1.45  (0.93-2.26) | 1.43  (0.86-2.39) | 1.08  (0.80-1.45) | 1.00  (0.73-1.37) |
| Non-anal STI in past 6 months | **2.42**  (1.59-3.68) | **1.88**  (1.20-2.94) | 1.01  (0.48-2.12) | 1.05  (0.45-2.45) | 0.81  (0.40-1.66) | 1.07  (0.49-2.37) | 1.34  (0.88-2.03) | 1.19  (0.75-1.91) |

Notes. Visits with missings were excluded (n=438, 6%). Hazard ratios are calculated relative to staying at the same risk level. Hazard ratios are shown in bold when the p-value is smaller than 0.05.

Abbreviations: CI=confidence interval; HR=hazard ratio; STI=sexually transmitted infection.
